# The Complete Mitochondrial Genome Sequence of *Eimeria kongi* (Apicomplexa: Coccidia)

**DOI:** 10.3390/life14060699

**Published:** 2024-05-29

**Authors:** Yubo Shi, Sufang Fang, Xiaolong Gu, Chengyu Hao, Fangchen Du, Ping Cui, Xinming Tang

**Affiliations:** 1 College of Animal Science and Technology, Hebei North University, Zhangjiakou 075000, China; shiyb0123@foxmail.com (Y.S.); zjkfsf@126.com (S.F.); bingli2006@126.com (X.G.); 17360708807@163.com (C.H.); dufangchen@gmail.com (F.D.); 2 Key Laboratory of Animal Biosafety Risk Prevention and Control (North), Key Laboratory of Veterinary Biological Products and Chemical Drugs of MARA, Institute of Animal Science, Chinese Academy of Agricultural Sciences, Beijing 100193, China

**Keywords:** rabbit coccidiosis, *Eimeria kongi*, mitochondrial genome, phylogenetic analyses

## Abstract

Rabbit coccidiosis is caused by infection with one or, more commonly, several *Eimeria* species that parasitize the hepatobiliary ducts or intestinal epithelium of rabbits. Currently, there are eleven internationally recognized species of rabbit coccidia, with the complete mitochondrial (mt) genomes of six species commonly infecting rabbits having been sequenced and annotated. *Eimeria kongi* was initially discovered in 2011 and prompted a preliminary study on this species. Through traditional morphological analysis, *E. kongi* was identified as a novel species of rabbit coccidia. To further validate this classification, we sequenced and annotated its mitochondrial genome. The complete mt genome of *E. kongi* spans 6258 bp and comprises three cytochrome genes (*cytb, cox1, cox3*), fourteen gene fragments for the large subunit (LSU) rRNA, and nine gene fragments for the small subunit (SSU) rRNA, lacking transfer RNA (tRNA) genes. Moreover, phylogenetic analysis of the mitochondrial genome sequence of *E. kongi* revealed its clustering with six other species of rabbit coccidia into a monophyletic group. Additionally, *E. irresidua* and *E. flavescens* were grouped within the lineage lacking oocyst residuum, consistent with their morphological characteristics. Consistent with multiple molecular phylogenies, in this investigation, *E. kongi* was further confirmed as a new species of rabbit coccidia. Our research findings are of great significance for the classification of coccidia and for coccidiosis prevention and control in rabbits.

## 1. Introduction

Rabbit coccidiosis is a parasitic disease caused by one or various species of *Eimeria*, which parasitize the bile ducts of the liver and the intestinal epithelial cells of rabbits. Internationally, there are 11 recognized species of coccidia that affect rabbits: *Eimeria stiedae*, *E. vejdovskyi*, *E. flavescens*, *E. media*, *E. intestinalis*, *E. perforans*, *E. piriformis*, *E. coecicola*, *E. exigue*, *E. magna*, and *E. irresidua* [1]. In a study conducted in 2011 by Ping Cui and colleagues in Hebei Province, a new species of rabbit coccidia, *Eimeria kongi*, was discovered and named [2]. Subsequent research focused on the morphological characteristics and lifecycle of *E. kongi*, utilizing sequencing of the 18S rRNA gene and the first internal transcribed spacer (ITS-1) for phylogenetic analysis. Although *E. kongi* was initially identified as a new species of rabbit coccidia, further evidence is required to conclusively establish it as a distinct coccidian species.

Mitochondria play a crucial role in energy metabolism in eukaryotic organisms, supplying energy for various vital processes. These organelles possess their own genome known as mitochondrial DNA (mtDNA). In comparison to the mtDNA of other organisms, the mitochondrial DNA of protists typically exhibits a linear structure and is notably shorter in length, with some species having genomes as compact as 6 kb, as seen in *Eimeria* [3,4,5,6,7,8,9]. Furthermore, the mitochondrial genomes of protists are characterized by their compact nature, containing a high density of genes with minimal non-coding regions. This results in small, sparse intervals between genes [10,11]. Serving as an independent repository of genetic information, mitochondrial genomes are inherited maternally, evolve rapidly, and lack recombination. Consequently, they are frequently employed as genetic markers with significant applicability in studies related to population genetics, ecological genetics, phylogeny, and evolutionary analysis [12,13,14,15,16].

In our previous studies, we have conducted research on the pathogenicity, immunogenicity, endogenous development, and drug resistance of *E. kongi*. Through this research, we have gained a preliminary understanding of its biological characteristics and conducted phylogenetic analysis using 18S rRNA [2,17]. However, other studies have shown that while the use of the 18S rRNA gene can generally aid in the classification of monophyletic groups, it may be insufficient for closely related species. Astrid M Tenter shares a similar perspective, suggesting that phylogenetic analysis based on a single molecular sequence of *Eimeria* may only represent the evolutionary history of that gene itself [18]. To establish the evolutionary relationships of *Eimeria* species accurately, it is preferable to analyze the phylogenetic consistency of multiple molecular sequences, ideally including sequences from both the nuclear and organelle genomes [19,20,21,22]. Therefore, in this study, we have sequenced the mitochondrial DNA of *E. kongi* and conducted a phylogenetic analysis with other reported *Eimeria* mtDNAs. The primary objectives of this study were to further verify the classification of *E. kongi* as a new species of rabbit coccidia using a molecular biology approach and to provide new mitochondrial genome data of rabbit coccidia for future research in molecular epidemiology, population genetics, and related fields.

## 2. Materials and Methods

### 2.1. Parasites

*Eimeria kongi* was originally isolated from rabbit fecal samples and has been meticulously conserved by the Animal Parasitology Laboratory of Hebei North University in Zhangjiakou, Hebei Province, China, since the year 2011. To maintain its viability, the parasite undergoes a regular rejuvenation process every six months.

### 2.2. Genomic DNA Extraction and PCR Amplification

#### 2.2.1. Oocyst Purification

The potassium dichromate in the suspension of *E. kongi* oocysts was removed by centrifugation at 1500× *g* for 5 min. Subsequently, three times the volume of 7.5% sodium hypochlorite (Sangon Co., Ltd., Shanghai, China) was added to the precipitate, and the mixture was allowed to react on ice for 3–5 min before another centrifugation at 1500× *g* for 5 min. Finally, the supernatant was transferred to a new centrifuge tube, and the sodium hypochlorite was washed away with PBS by centrifugation at 1500× *g* for 5 min.

#### 2.2.2. Genomic DNA Extraction

A total of 2 × 10^7^ purified *E. kongi* oocysts were disrupted using 1 mm diameter glass beads. The suspension containing sporocyst was centrifuged to obtain the precipitate. To this precipitate, 500 μL of CTAB solution (Solarbio Co., Ltd., Beijing, China) and 40 μL of proteinase K (20 mg/mL) (Zoman Bio Co., Ltd., Beijing, China) were added, and the mixture was incubated in a water bath at 55 °C for 1 to 3 h. Subsequently, phenol:chloroform:isoamyl alcohol (25:24:1) (Solarbio Co., Ltd., Beijing, China) was added to remove impurities such as proteins. An equal volume of isopropanol was then added to precipitate the DNA. Finally, the DNA was washed with 70% ethanol to obtain high-purity *E. kongi* genomic DNA, which was dissolved in double-distilled water and stored at −20 °C until use.

#### 2.2.3. PCR Amplification

We utilized the extracted *E. kongi* oocyst DNA as a template for nested PCR utilizing LA Taq polymerase (Takara Bio Co., Ltd., Beijing, China) following the manufacturer’s instructions. Two sets of primers were designed based on the documented mitochondrial genome sequences of six rabbit coccidia to amplify the mtDNA of *E. kongi*.

The first primer set consists of Emtk1-F (5′-TATTCCTATTAGCACAATCTTTCTT-3′) and Emtk1-R (5′-CAATCCTTTTCCATCTGTTCTT-3′).

The second primer set comprises Emtk2-F (5′-CTCATTCTTTCTTTGGTCTA-3′) and Emtk2-R (5′-TCCCAACCGAAAGACACCT-3′).

The first round of the PCR reaction, with a total volume of 50 μL, consists of the following components: 0.5 μL LA Taq (5 U/μL), 5 μL 10× LA Taq PCR Buffer, 8 μL dNTP Mixture (2.5 mM each), 1 μL *E. kongi* genomic DNA (500 ng/μL), 2 μL Emtk1-F primer (10 μM), 2 μL Emtk1-R primer (10 μM), and double-distilled water to bring the final volume to 50 μL.

The cycling conditions for the first round of PCR are as follows: initial denaturation at 94 °C for 10 min, followed by 30 cycles of denaturation at 94 °C for 30 s, annealing at 56 °C for 30 s, extension at 72 °C for 6 min, and a final extension at 72 °C for 10 min.

Compared with the first round of the PCR system, the second round only changed the template from genomic DNA to the first round of PCR product.

The conditions for the second round of PCR were the same as in the first round.

The resultant product from nested PCR was sequenced and assembled by Sangon Co., Ltd. (Shanghai, China) using next-generation sequencing techniques.

### 2.3. Genome Annotation and Sequence Analysis

The mtDNA sequence of *E. kongi* was analyzed using SnapGene v6.0.2 and compared with the mtDNA sequences of 18 other reported *Eimeria* species. This analysis delineated the genome structure and protein-coding genes’ boundaries. The entire *E. kongi* mtDNA sequence, spanning 6258 bp, has been deposited in GenBank under accession number NC_072899.

### 2.4. Clustal Alignment and Phylogenetic Analyses

To investigate the phylogenetic relationship between *E. kongi* and other species of *Eimeria*, we aligned the mtDNA sequence of *E. kongi* with that of 18 *Eimeria* species from GenBank using ClustalX v2.1 (Table 1). Subsequently, a Neighbor-Joining tree was constructed using MEGA v10.2.6, and bootstrap analysis was performed with 1000 replicates.

## 3. Results and Discussion

### 3.1. Analyzing the mtDNA Composition of E. kongi

**Figure 1:**
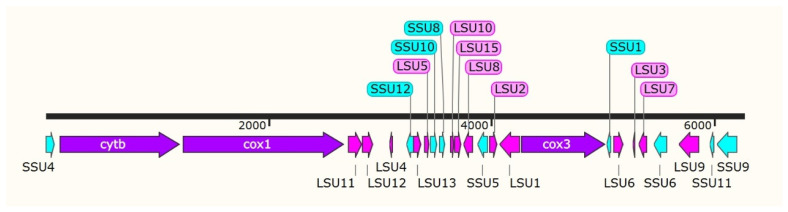
Structure of the mtDNA of *E. kongi*. The spatial arrangement, length, and transcriptional orientation of genes within the mtDNA of *E. kongi* were elucidated through comparative analysis with the mitochondrial genomes of six rabbit coccidian species using Clustal v2.1. This comparative approach facilitated the prediction of gene locations within the *E. kongi* mitochondrial genome. In the resulting schematic representation, protein-coding genes are denoted in purple, fragments of LSU rRNA are highlighted in pink, and fragments of SSU rRNA are depicted in blue. The transcriptional direction of each gene is graphically represented by arrows at the respective gene termini.

In the existing literature, the mitochondrial genomes of apicomplexan protozoa, which include species such as *Toxoplasma*, *Cyclospora*, *Isospora*, and *Eimeria*, are relatively compact, averaging around 6 kilobases (kb) in size [23,24,25,26,27,28]. These organisms not only share a similar gene composition but they also exhibit a conserved gene arrangement. Specifically, they contain three cytochrome genes (*cox1*, *cox3*, *cytb*), as well as genes for the large- and small-subunit ribosomal RNAs (LSU and SSU rRNAs, respectively). Building upon this foundation, the present study aimed to annotate the mitochondrial genome of *E. kongi* by examining the complete mtDNA sequence. This was achieved through a comparative analysis of the gene content and genome architecture with 18 strains of *Eimeria* [29,30]. The complete mtDNA sequence of *E. kongi* spans 6258 bp and includes 3 protein-coding genes (*cytb*, *cox1*, and *cox3*), 14 fragments of the LSU rRNA gene, and 9 fragments of the SSU rRNA gene. Notably, the sequence lacks any transfer RNA (tRNA) genes, aligning with the characteristics observed in *Eimeria* species isolated from rabbits (Figure 1).

The *cytb* in *E. kongi* mtDNA spans a length of 1080 bp, located between nucleotide positions 128 and 1207, and initiates with the start codon ATG. The cytochrome c oxidase subunit I gene (*cox1*) extends over 1443 bp, ranging from nucleotide 1240 to 2682, also beginning with the ATG start codon. The *cox3* is 756 bp in length, positioned between nucleotides 4279 and 5034, and it begins with the less common start codon TTA. Notably, all three protein-coding genes share the TAA stop codon (Table 2). Furthermore, the inferred translational direction for these genes—*cytb*, *cox1*, and *cox3*—conforms to the orientation observed in *Eimeria* species isolated from domestic rabbits (Figure 1).

The mitochondrial genome of *E. kongi* exhibits a pronounced adenine and thymine (A + T) content of 65.42%, with a corresponding guanine and cytosine (G + C) content of 34.58%. This can be further broken down into individual nucleotide frequencies: 29.80% adenine (A), 35.62% thymine (T), 16.95% guanine (G), and 17.63% cytosine (C). The sequence of *E. kongi*’s mtDNA offers novel genetic markers that are valuable for the study of molecular epidemiology and population genetics within the *Eimeria* genus. Moreover, it holds potential for advancing the molecular diagnosis and management strategies for rabbit coccidiosis. Among the 19 recognized species of *Eimeria*, the mtDNA lengths vary, with *E. mitis* featuring the longest mtDNA at 6408 bp, while *E. brunetti* has the shortest at 6156 bp. A notable characteristic across all *Eimeria* species is their high A + T content, which exceeds 60%. When comparing the nucleotide compositions, the six species of rabbit coccidia, particularly *E. irresidua*, show the closest similarity to *E. kongi* (Table 3).

### 3.2. Clustal Alignment and Phylogenetic Analysis

**Figure 2:**
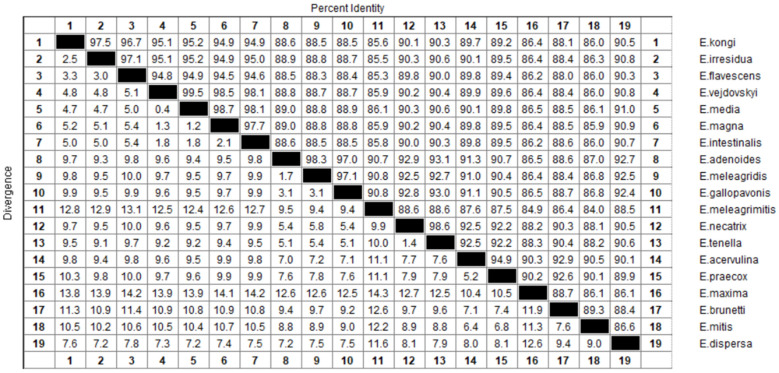
The nucleotides sequences of the complete mtDNA among the 18 *Eimeria* species and *E. kongi*.

The complete mitochondrial genome sequence of *E. kongi* was compared with those of 18 other *Eimeria* species, as shown in Figure 2. The sequence identity between *E. kongi* and rabbit coccidia species ranged from 94.9% to 97.5%, with *E. irresidua* and *E. flavescens* showing the highest identities at 97.5% and 96.7%, respectively. In contrast, other chicken and turkey coccidia species exhibited less than 90.5% sequence identity with *E. kongi*. Among these, *E. meleagrimitis* had the lowest identity at 85.6%, while *E. dispersa* had the highest at 90.5%.

**Figure 3:**
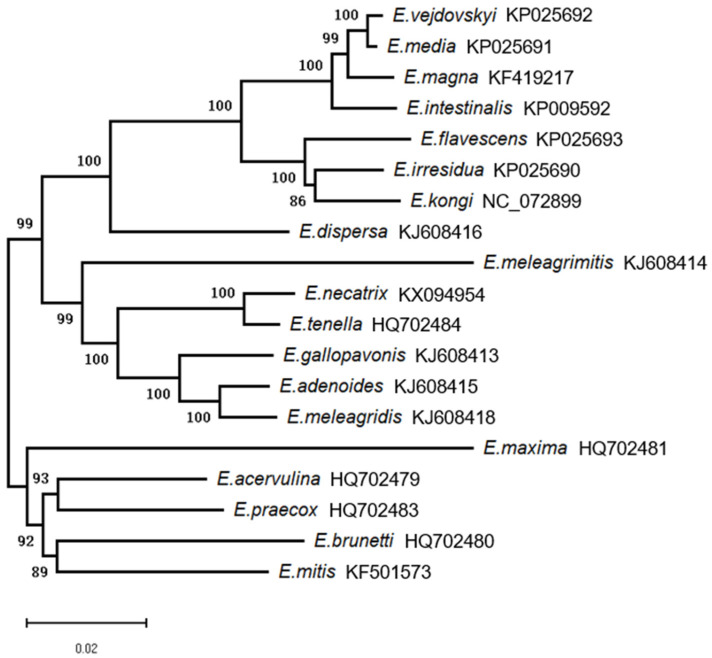
Phylogenetic analysis of *E. kongi* was conducted utilizing mtDNA sequences. The analysis employed the Neighbor-Joining (NJ) method, and nodes with percentage support exceeding 50% from 1000 pseudoreplicates are marked on the left side of the corresponding nodes.

As depicted in Figure 3, phylogenetic analysis revealed a pattern of clustering among certain *Eimeria* species that is congruent with their host organisms. Specifically, a monophyletic group encompasses seven species of rabbit coccidia, including *E. kongi*. However, this host-based clustering cannot be observed uniformly across all *Eimeria* species; for instance, chicken and turkey coccidia do not form such distinct host-related clusters. Among the chicken coccidia, *E. necatrix* and *E. tenella*, along with the turkey coccidia *E. meleagrimitis*, *E. adenoides*, *E. gallopavonis*, and *E. meleagridis*, form one monophyletic group. Conversely, the remaining five species of chicken coccidia—*E. maxima*, *E. acervulina*, *E. praecox*, *E. brunetti*, and *E. mitis*—constitute another monophyletic group. Interestingly, *E. dispersa* is not categorized within the previously mentioned monophyletic groups; instead, it is found to be closely related to the rabbit coccidia, belonging to the same monophyletic group. Furthermore, the monophyletic group of rabbit coccidia can be differentiated into two distinct sister lineages based on the presence or absence of oocyst residuum. *E. kongi*, *E. irresidua*, and *E. flavescens* are grouped within the lineage characterized by the absence of oocyst residuum. In contrast, *E. magna*, *E. intestinalis*, *E. media*, and *E. vejdovskyi* are classified within the lineage that exhibits oocyst residuum.

In summary, the phylogenetic tree derived from mtDNA sequences was found to be congruent with the trees constructed using 18S rRNA and ITS-1 sequences, as reported by Ping Cui and colleagues [2]. This concordance substantiates the classification of *E. kongi* within a monophyletic group alongside other species responsible for rabbit coccidiosis. Furthermore, we surmise that the oocyst residuum, a key morphological characteristic traditionally used in the taxonomy of rabbit coccidia, represents a phenotypic trait that arises from the gene expression of these organisms. Its significance in classification is as reliable as molecular markers. *E. kongi* is grouped with species that lack an oocyst residuum within the rabbit coccidia monophyletic group, a classification that aligns with its morphological features. The phylogenetic analysis based on multiple molecular sequences for *E. kongi* is consistent, and this genetic evidence, in conjunction with the organism’s morphological traits, strongly supports the designation of *E. kongi* as a distinct species within the rabbit coccidia, rather than a mere counterpart of existing species.

## 4. Conclusions

This study has elucidated that the mitochondrial genome of *E. kongi* is a linear DNA molecule, with a total length of 6258 bp. The nucleotide composition, as well as the directionality of protein translation and the utilization of start and stop codons within the *E. kongi* mitochondrial genome, closely resemble those observed in other *Eimeria* species’ mitochondrial genomes. Moreover, the phylogenetic analysis conducted using the complete mitochondrial genome sequence identified *E. kongi* as a novel species of rabbit coccidia endemic in China. It is positioned within a distinct sister lineage characterized by the absence of oocyst residuum, within the monophyletic group of rabbit coccidia. Ultimately, the mtDNA sequence of *E. kongi* offers novel DNA markers that will be instrumental for the investigation of molecular epidemiology and population genetics specific to this species. Furthermore, the mtDNA sequence aids in the molecular diagnosis of rabbit coccidiosis. Overall, the comprehensive mtDNA dataset serves as a valuable resource, providing essential information for phylogenetic analysis and population genetics research across various parasite populations.

## Figures and Tables

**Table 1:** GenBank accession numbers for the 19 complete mtDNA of *Eimeria* species in this study.

Species	Accession Number	Species	Accession Number
*E* *. kongi*	NC_072899	*E* *. gallopavonis*	KJ608413
*E* *. vejdovskyi*	KP025692	*E* *. meleagrimitis*	KJ608414
*E* *. media*	KP025691	*E* *. necatrix*	KX094954
*E* *. magna*	KF419217	*E* *. tenella*	HQ702484
*E* *. intestinalis*	KP009592	*E* *. acervulina*	HQ702479
*E* *. flavescens*	KP025693	*E* *. praecox*	HQ702483
*E* *. irresidua*	KP025690	*E* *. maxima*	HQ702481
*E* *. dispersa*	KJ608416	*E* *. brunetti*	HQ702480
*E* *. adenoides*	KJ608415	*E* *. mitis*	KF501573
*E* *. meleagridis*	KJ608418		

**Table 2:** Complete mitochondrial genome of *E. kongi*.

Genes	Nucleotide No.	Size (bp)	Start Codon	Stop Codon
SSU4	1–82	82		
cytb	128–1207	1080	ATG	TAA
cox1	1240–2682	1443	ATG	TAA
LSU11	2722–2849	128		
LSU12	2851–2949	99		
LSU4	3090–3114	24		
SSU12	3244–3304	61		
LSU13	3305–3379	75		
LSU5	3403–3451	49		
SSU10	3460–3524	65		
SSU8	3538–3596	59		
LSU10	3637–3669	33		
LSU15	3678–3733	56		
LSU8	3757–3837	81		
SSU5	3883–3974	92		
LSU2	3992–4057	66		
LSU1	4083–4261	179		
cox3	4279–5034	756	TTA	TAA
SSU1	5044–5073	30		
LSU6	5102–5195	94		
LSU3	5272–5295	24		
LSU7	5331–5403	73		
SSU6	5467–5582	116		
LSU9	5684–5871	188		
SSU11	5970–6006	37		
SSU9	6032–6212	181		

**Table 3:** Nucleotide composition of the mitochondrial genome of the 19 *Eimeria* species.

Species	A (%)	T (%)	G (%)	C (%)	Total
*E*. *mitis*	30.9	36.5	16.3	16.3	6408
*E*. *irresidua*	29.8	35.6	17	17.6	6259
*E*. *kongi*	29.8	35.6	17	17.6	6258
*E*. *flavescens*	30	35.5	16.9	17.6	6258
*E*. *magna*	29.7	35.4	17.1	17.7	6249
*E*. *intestinalis*	29.7	35.5	17.1	17.7	6247
*E*. *media*	29.6	35.5	17.1	17.7	6245
*E*. *vejdovskyi*	29.6	35.5	17.2	17.7	6240
*E*. *dispersa*	29.9	35.6	16.8	17.6	6238
*E*. *gallopavonis*	30.1	34.8	16.9	18.3	6215
*E*. *tenella*	29.8	35.2	16.9	18.1	6213
*E*. *meleagridis*	29.8	34.9	17	18.3	6212
*E*. *necatrix*	29.8	35.2	16.9	18.2	6212
*E*. *adenoides*	29.8	34.8	17	18.4	6211
*E*. *acervulina*	30.1	35.3	17	17.6	6179
*E*. *praecox*	30.1	35.5	17.1	17.3	6172
*E*. *maxima*	30	34.5	17.5	17.9	6167
*E*. *meleagrimitis*	30	33.5	17.3	19.2	6165
*E*. *brunetti*	29.8	35.8	17.1	17.4	6156

## Data Availability

The genome sequences are available in the NCBI, and Table 1 has listed all strains’ accession numbers.
